# Association between telomere length and hepatocellular carcinoma risk: A Mendelian randomization study

**DOI:** 10.1002/cam4.5702

**Published:** 2023-03-07

**Authors:** Chenglei Yang, Xi Wu, Siyu Chen, Bangde Xiang

**Affiliations:** ^1^ Department of Hepatobiliary Surgery Guangxi Medical University Cancer Hospital Nanning Guangxi P.R. China; ^2^ The First Clinical Medical School Guangxi Medical University Nanning Guangxi P.R. China

**Keywords:** genome‐wide association study, hepatocellular carcinoma, Mendelian randomization, single nucleotide polymorphism, telomere length

## Abstract

**Background:**

Hepatocellular carcinoma (HCC) is a common cancer threatening the public health globally. Although HCC has been associated with the telomere length (TL), the causal relationship between them is not well understood. Therefore, we attempted to explore the linear causal relationship between TL and HCC through Mendelian randomization (MR) analysis among Asian and European populations.

**Methods:**

The summary statistics of TL‐associated single nucleotide polymorphisms (SNPs) were obtained from a genome‐wide association study (GWAS) in the Asian population (*N* = 23,096). The data of TL‐associated SNPs in the European population (*N* = 472,174) and the GWAS summary statistics of HCC in the Asian population (1866 cases, 195,745 controls) as well as the European population (168 cases, 372,016 controls) were downloaded from the public GWAS database. Two‐sample MR was performed using inverse variance weighting (IVW), weighted median estimate, MR–Egger regression, weighted‐mode estimate, and simple‐mode estimate methods. Sensitivity analysis was performed to text the primary results' robustness.

**Results:**

Nine SNPs associated with TL in Asian populations and 98 SNPs in European populations were selected as instrumental variables. No linear causal relationship between heritable TL and the HCC risk was recorded in the Asian (IVW analysis odds ratio [OR] = 1.023, 95% confidence interval [CI] 0.745, 1.405, *p* = 0.887) and European populations (IVW analysis OR = 0.487, 95% CI 0.180, 1.320, *p* = 0.157). Other methods also achieved similar outcomes. Sensitivity analysis was performed and revealed no heterogeneity and horizontal pleiotropy.

**Conclusions:**

No linear causal association was recorded between heritable TL and HCC in Asian and European populations.

## Graphical abstract

elomere length and hepatocellular carcinoma in Asian and European populations using mendelian randomization.

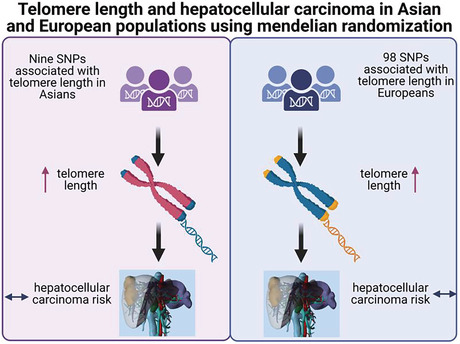

## 1 INTRODUCTION

Hepatocellular carcinoma (HCC), the most common type of liver cancer, ranks fourth among the most common causes of cancer‐related death in the world, seriously endangering health.^1^ Owing to the varied heterogeneity in the pathogenesis and histopathology of HCC, the patients present with poor prognoses. HCC imposes enormous pain and a huge financial burden on the patient. Currently, hepatitis B and C are the leading risk factors for HCC. Furthermore, metabolic syndrome, obesity, and excessive alcohol consumption are known to enhance HCC risk.^2^ In addition, genetic factors play an important role in HCC development. Numerous HCC genetic‐susceptibility variations have been effectively reported by genome‐wide association studies (GWASs). Among the susceptibility variants, *PNPLA3*, *SAMM50*, and *TM6SF2* variants are considered as risk factors for HCC, while locus rs2242652 in *TERT* served as a protective factor.^3^, ^4^, ^5^

Telomeres are DNA‐protein complexes located at the ends of eukaryotic chromosomes and consist of a tandem repeat nucleotide sequence (TTAGGG) and wrapped by a cap‐like protein complex called “shelterin”.^6^ Normally, only pluripotent stem cells and the early stages of embryonic growth display a high expression of telomerase to resist telomere attrition. Moreover, the telomere length (TL) generally shortens with physiological aging in humans.^7^ Shortened TL can induce cell senescence or apoptosis, which inhibits tumorigenesis. However, it can also promote the accumulation of genomic instability through complex chromosomal rearrangements such as aneuploidy and gene amplification, ultimately leading to continued malignant cell division.^8^ On the contrary, some studies have linked long TL with a higher cancer risk, such as lung cancer and non‐Hodgkin lymphoma.^9^, ^10^ Recent studies have demonstrated an association of TL with the risk of HCC.^11^, ^12^, ^13^ Until date, only one research explored the causal relationship between TL and hepatitis B virus (HBV)‐related HCC in the Asian population.^14^ The causal relationship between TL and HCC of unknown causes is, however, unclear. Therefore, it is crucial to clarify the risk factors of HCC by analyzing multiple populations to comprehend the causal relationships toward promoting its prevention.

**FIGURE 1:**
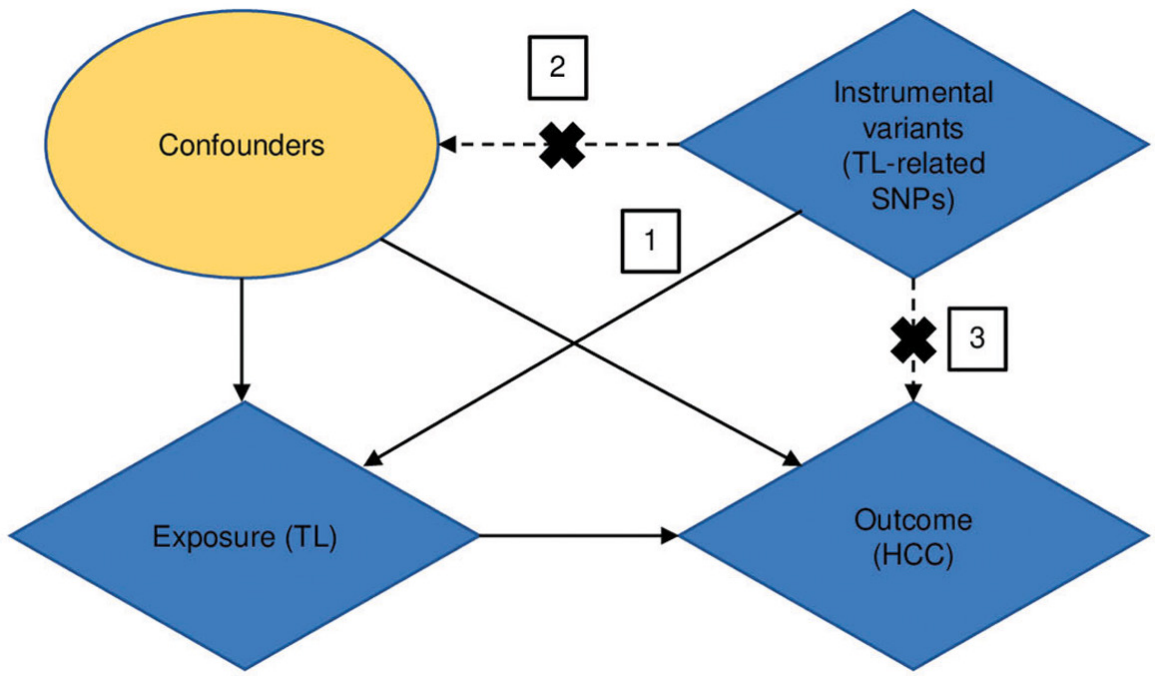
Schematic representation of the two‐sample MR analysis. Number 1 represents that instrumental variables (IVs) are strongly associated with exposure and only affected the outcome through the exposure; number 2 represents that IVs are not associated with confounders; number 3 represents that IVs are not directly associated with the outcome. HCC, hepatocellular carcinoma; MR, Mendelian randomization; TL, telomere length.

Mendelian randomization (MR) analysis is an epidemiological study that explores whether a specific factor (exposure) is causally related to a certain disease (outcome). While exploring the causality, MR uses single nucleotide polymorphisms (SNPs) associated with factors such as instrumental variables (IVs) and is usually unaffected by confounding and reverse causality that are commonly observed in observational studies.^15^, ^16^ MR analysis is based on the following three assumptions: (1) genetic variants as IVs are robustly associated with exposure (TL), (2) IVs are not associated with confounders in the exposure–outcome relationship, and (3) genetic variant is associated with the outcome (HCC) via exposure (TL) (Figure 1).^17^ Previously, the GWASs performed in the Asian and European populations have identified several SNPs associated with TL or HCC. This observation further enabled us to conduct MR analysis toward exploring the causal relationship between TL and HCC.

Our study evaluated the linear causal relationship between TL and HCC by using SNPs that are robustly associated with TL as IVs in Asian and European populations through a two‐sample MR analysis.

## 2 MATERIALS AND METHODS

### 2.1 Data sources

The summary statistics of SNPs were obtained from a meta‐GWAS that identified nine SNPs in the linkage equilibrium with significantly associated with TL in the Asian population (*N* = 23,096).^18^ The data of SNPs associated with TL in the European population (*N* = 472,174; study ID “ieu‐b‐4879”) and the GWAS summary statistics of HCC in the Asian population (1866 cases, 195,745 controls; study ID “bbj‐a‐158”), and the European population (168 cases, 372,016 controls; study ID “ieu‐b‐4953” were downloaded from the publicly available IEU GWAS database; https://gwas.mrcieu.ac.uk/datasets/). The quality‐control procedure undertaken and the pipeline of HCC GWAS produced by the IEU GWAS database associated with the full UK Biobank has been described in detail elsewhere.^19^, ^20^, ^21^

### 2.2 SNP selection

To satisfy the three assumptions of MR analysis, a series of strict quality‐control measures were practiced for selecting SNPs. Among Asians, we first adopted a strict threshold of genome‐wide significance (*p* < 5 × 10^−8^) as the inclusion criteria. In order to accurately identify the effect alleles in the exposure and outcome GWAS datasets, palindromic SNPs, representing alleles with the same pair of letters (i.e., A/T or G/C) in the forward and reverse strands of DNA double‐helix structure, with allele frequency close to 50% were pruned.^22^, ^23^ Second, the PhenoScanner tool (http://www.phenoscanner.medschl.cam.ac.uk/) was employed to detect pleiotropic SNPs associated with known HCC risk factors, such as hepatitis B, cirrhosis, obesity, and prune, if present.^24^ Third, to avoid bias induced by weak IVs, we calculated the F statistics using the following formula: *F* = *R* ^2^ (*n* − *k* − *1*)/*k* (*1* − *R* ^2^), where, *R* ^2^, *n*, and *k* represent the TL variation explained by IVs, the sample size, and the IVs numbers, respectively.^24^, ^25^ *R* ^2^ can be calculated by using the following formula: 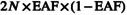 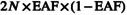.^26^ EAF, *β*, *N*, and se(*β*) represent the effect allele frequency, the estimated genetic effect on TL, the sample size, and the standard error, respectively. If the *F*‐statistic was >10, we considered it to be less likely to be weak IV. Next, the information on these SNPs of HCC from the IEU GWAS dataset was extracted. Among Europeans, particularly, we converted the bolt‐linear mixed model effect sizes and SE of HCC statistical data into an approximate OR scale using a Taylor transformation expansion series.^27^, ^28^ First, the inclusion criteria were kept the same as that for the Asians (*p* < 5 × 10^−8^). Second, the SNPs in linkage disequilibrium (*r* ^2^ < 0.001, window size = 10,000 kb) were removed. Then, we detected pleiotropic SNPs, identified palindromic SNPs, calculated *F* statistics, and extracted the corresponding HCC data using the same methods as for the Asian population.

### 2.3 MR analysis

We combined the summary statistics (βSNP–TL and βSNP–HCC) in Asians and Europeans, respectively, to estimate the linear causal relationship between heritable TL and the risk of HCC. The inverse variance weighting (IVW) is an effective analysis under the assumption that all genetic variants are valid IVs, specifically requiring that genetic variants affect the target outcome (HCC) only through the pathway by which exposure (TL) affects the outcome (HCC) (i.e., no horizontal pleiotropy).^17^ We adopted four additional methods, including MR–Egger regression, weighted median estimate, weighted‐mode estimate, and simple‐mode estimate that are based on different assumptions. Even if all IVs were invalid, MR–Egger regression provided an effect estimate consistent with the true effect.^29^ The linear causal relationship between TL and HCC was biased in the case of a horizontal pleiotropy, but when 50% of the SNPs were valid instruments, the weighted median estimate could yield estimates consistent with the final effect.^30^ Weighted‐mode estimate and simple‐mode estimate relaxed the assumption of IVs and promoted the reliability and stability of the causal effect detection.^31^ The results are expressed as the odds ratios (ORs) and 95% confidence intervals (CIs) of HCC risk for a unit standard deviation (SD) change in TL.

### 2.4 Sensitivity analysis

The IVW analysis and MR–Egger regression were tested for heterogeneity. Cochran's *Q* test was applied to assess the heterogeneity of IVs, and *p* < 0.05 was considered significantly heterogeneous. If the genetic variants used as IVs are found to be associated with risk factors other than the exposure of interest and have a causal effect on the outcome, there are bias caused by horizontal pleiotropy; as such, the MR assumptions 2 and 3 are violated. MR–Egger regression can be applied to evaluate the bias caused by horizontal pleiotropy. When the intercept is close to 0 (*p* > 0.05), horizontal pleiotropy is considered absent.^32^ In addition, the leave‐one‐out method was followed in this analysis. Every SNP was removed sequentially, and the pooled effect of the remaining SNPs was calculated to assess whether there were potential SNPs with a driving effect on the causal effect.

### 2.5 Statistical analysis

All statistical analyses were performed in this study using the “Two‐sample MR” package in the R version 4.0.1, with a two‐sided *p* < 0.05 considered to indicate statistical significance.

## 3 RESULTS

**FIGURE 2:**
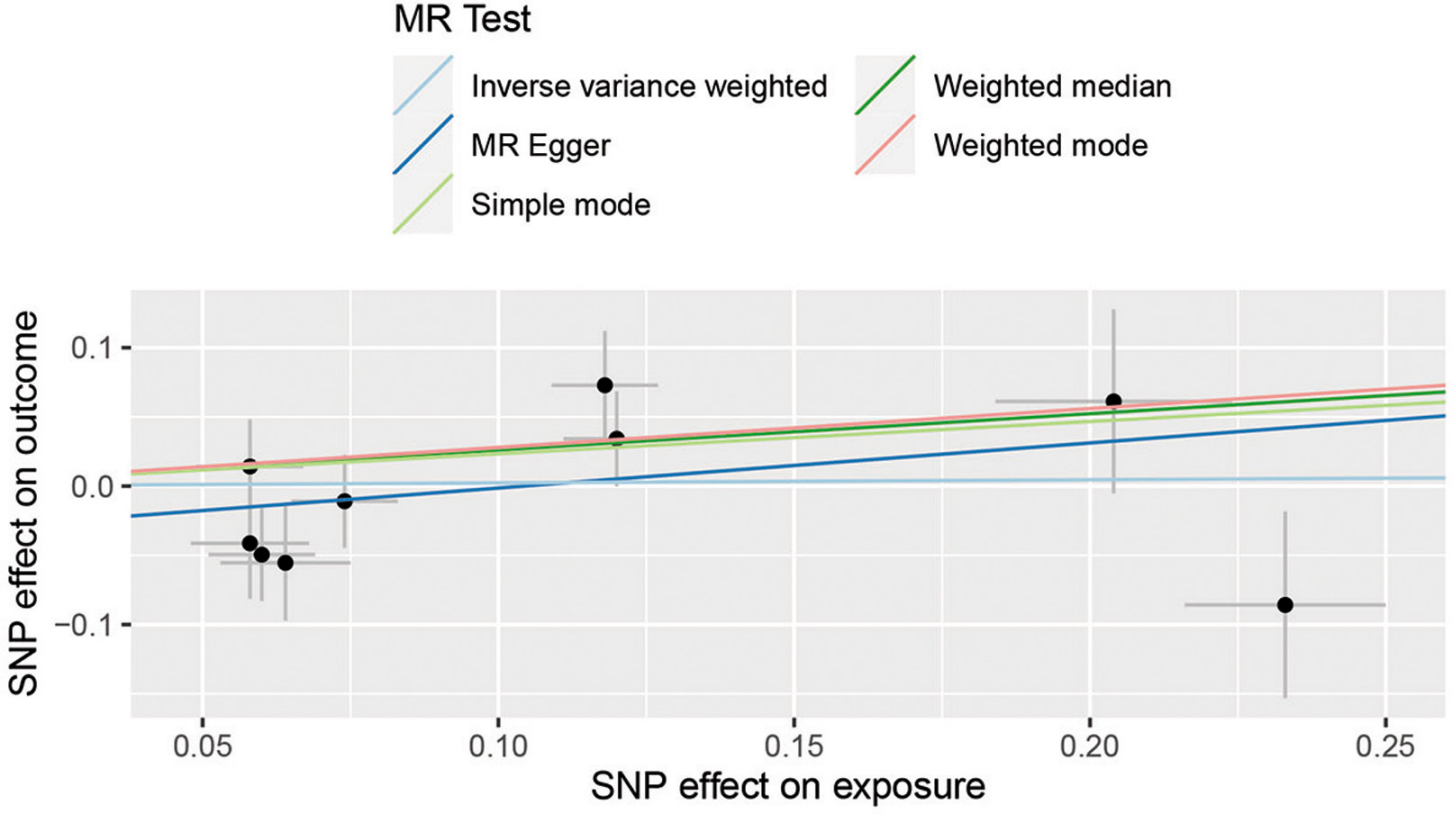
Scatter plots for MR analyses of the causal effect of TL‐related SNPs on HCC in Asians. Each point represents a single SNP, and the line on each point represents 95% CI. The slope of each line represents the MR effect for each method. Exposure, telomere length; outcome, hepatocellular carcinoma. CI, confidence interval; HCC, hepatocellular carcinoma; MR, Mendelian randomization; SNP, single nucleotide polymorphism; TL, telomere length.

**FIGURE 3:**
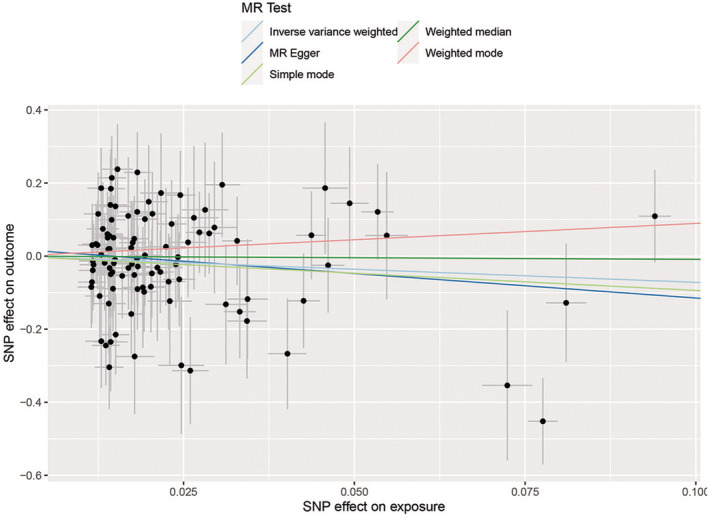
Scatter plots for MR analyses of the causal effect of TL‐related SNPs on HCC in the Europeans. Each point represents a single SNP, and the line on each point represents 95% CI. The slope of each line represents the MR effect for each method. Exposure, telomere length; outcome, hepatocellular carcinoma. CI, confidence interval; HCC, hepatocellular carcinoma; MR, Mendelian randomization; SNP, single nucleotide polymorphism; TL, telomere length.

A total of nine SNPs in Asian and 98 SNPs in European populations were included in this study. The characteristics of these SNPs and their effects associated with HCC are depicted in Table S1. These nine SNPs explain 3.71% of the variation in TL among Asian individuals and 98 SNPs explain 2.63% of the variation in TL among European population. The *F*‐statistics of all SNPs were >10. The MR analysis performed using the IVW analysis, weighted median estimate, MR–Egger regression, weighted‐mode estimate, and simple‐mode estimate revealed no significant evidence of a linear causal relationship between heritable TL and HCC in Asians (IVW analysis OR = 1.023, 95% CI 0.745, 1.405, *p* = 0.887) and Europeans (IVW analysis OR = 0.487, 95% CI 0.180, 1.320, *p* = 0.1574) (Table 1). Scatter plots demonstrated the effect of sizes of SNPs on exposure (TL) and outcome (HCC) in Asians (Figure 2) and Europeans (Figure 3).

**TABLE 1:** MR analysis for TL on HCC.

Population	Method	*N* SNPs	OR (95% CI)	*p*‐value
Asian	MR–Egger	9	1.384 (0.678, 2.828)	0.401
	Weighted median	9	1.298 (0.914, 1.846)	0.145
	Inverse‐variance weighted	9	1.023 (0.745, 1.405)	0.887
	Simple mode	9	1.262 (0.675, 2.361)	0.486
	Weighted mode	9	1.323 (0.779, 2.247)	0.330
European	MR–Egger	98	0.261 (0.042, 1.610)	0.151
	Weighted median	98	0.914 (0.191, 4.377)	0.911
	Inverse‐variance weighted	98	0.487 (0.180, 1.320)	0.157
	Simple mode	98	0.391 (0.013, 11.899)	0.586
	Weighted mode	98	2.445 (0.227, 26.316)	0.475

Abbreviations: CI, confidence interval; HCC, hepatocellular carcinoma; MR, Mendelian randomization; N SNPs, number of single nucleotide polymorphisms; OR, odds ratio; TL, telomere length.

**FIGURE 4:**
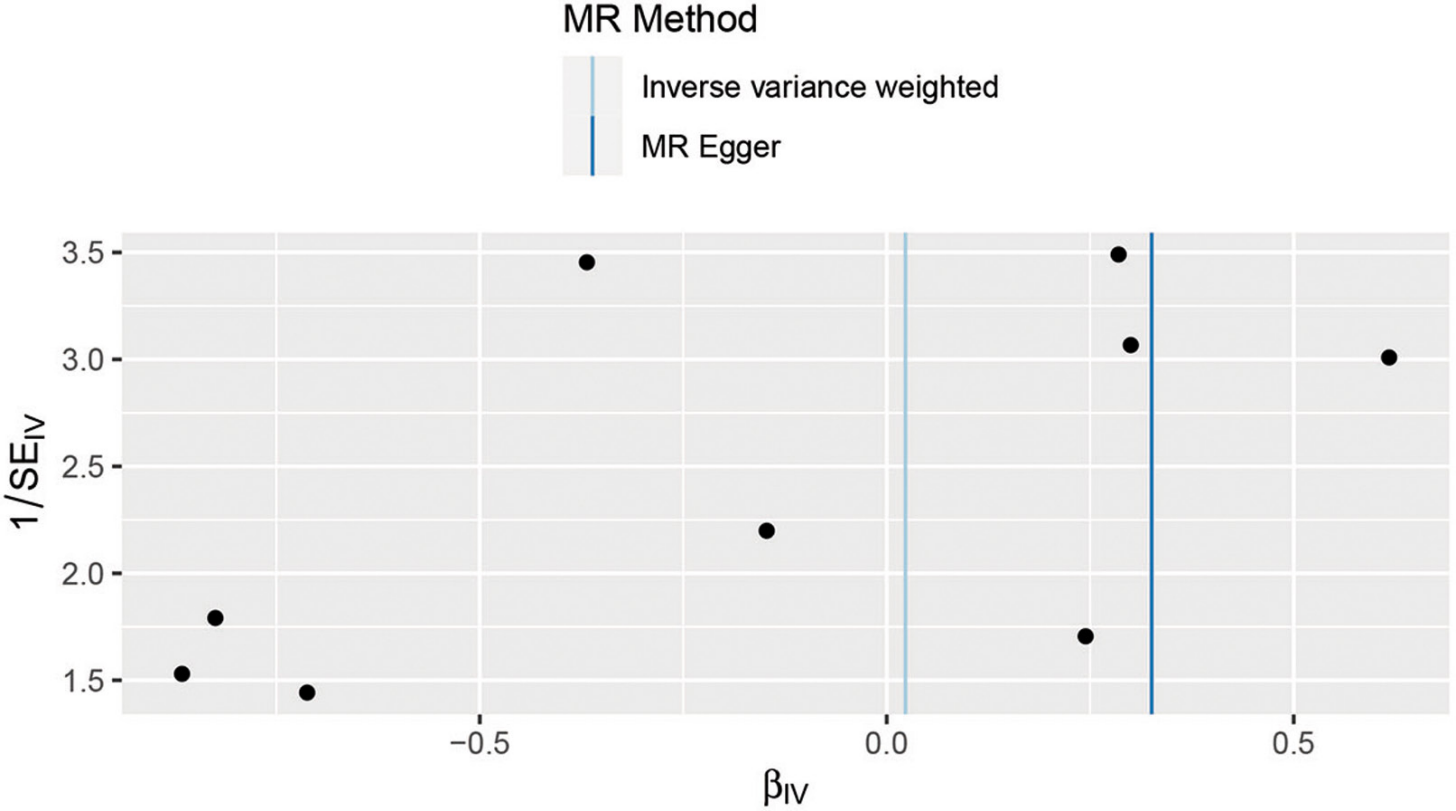
Funnel plot for heterogeneity test in the Asians. The vertical blue and dark blue lines represent the causal effects estimated using the inverse variance weighting and MR–Egger methods, respectively. IV, instrumental variable; MR, Mendelian randomization; SE, standard error.

**FIGURE 5:**
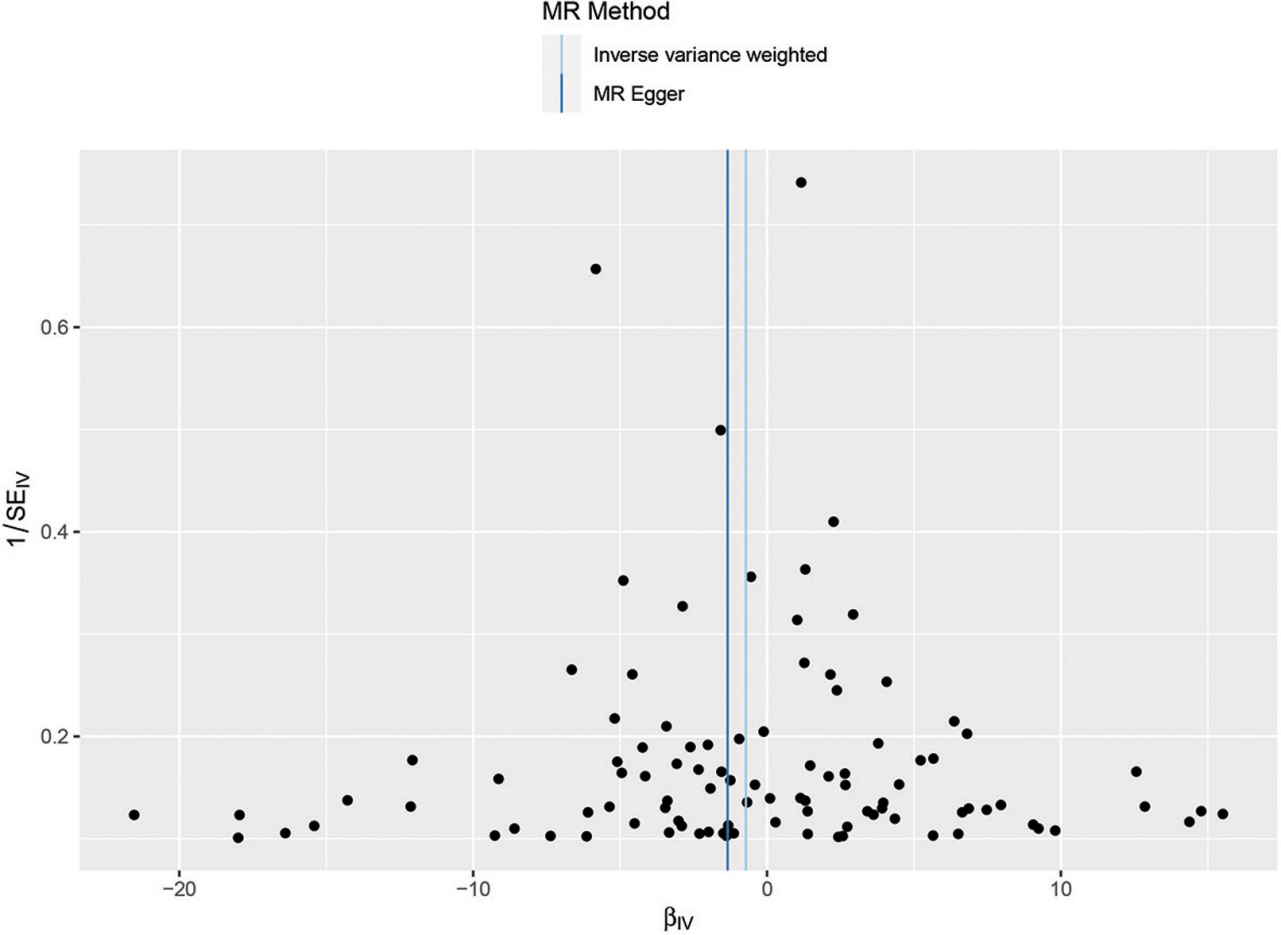
Funnel plot for heterogeneity test of single nucleotide polymorphisms in the Europeans. The vertical blue and dark blue lines represent the causal effects estimated using the IVW and MR–Egger methods, respectively. IV, instrumental variable; MR, Mendelian randomization; SE, standard error.

The results of IVW analysis and MR–Egger regression used for heterogeneity displayed insufficient evidence for heterogeneity among IVs in Asians [IVW, *Q* (df) 12.144 (8) *p* = 0.145; MR–Egger, *Q* (df) 10.811 (7), *p* = 0.147] and Europeans [IVW, *Q* (df) 104.419 (97), *p* = 0.285; MR–Egger, *Q* (df) 103.722 (96), *p* = 0.277]. Moreover, the obtained results were less likely to be affected by any potential bias (Table 2). The funnel plot displayed a symmetrical distribution of data, representing the causal association effects when a single SNP was used as the IV in Asians (Figure 4) and Europeans (Figure 5). This indicated that a potential bias was less likely to affect the causal association.

**TABLE 2:** Heterogeneity tests for IVs.

Population	MR method	Cochran *Q* statistics (df)	*p*‐value
Asian	MR–Egger	10.811 (7)	0.147
	Inverse‐variance weighted	12.144 (8)	0.145
European	MR–Egger	103.722 (96)	0.277
	Inverse‐variance weighted	104.419 (97)	0.285

Abbreviations: IV, instrumental variable; MR, Mendelian randomization.

The MR–Egger regression for IVs confirmed that no horizontal pleiotropy existed in Asians (intercept *β* = −0.034, *p* = 0.384) and Europeans (intercept *β* = 0.019, *p* = 0.424). Furthermore, the “leave‐one‐out” analysis was performed by eliminating each SNP sequentially, which suggested that no particular IV had a driving effect on the linear causal relationship in Asians (Figure S1) and Europeans (Figure S2).

## 4 DISCUSSION

We employed summary statistics of some GWASs to investigate the linear causal relationship between human heritable TL and the risk of HCC through a two‐sample MR analysis. No strong evidence was identified hinting a linear causal relationship between heritable TL and the risk of HCC in both Asian and European populations. In fact, subsequent sensitivity analyses supported these conclusions.

MR methods are based on the principle that genetic variants related to TL are randomly assigned to individual gametes at meiosis. Genetically predicted TL is determined at an individual's birth, chronologically before the onset of tumors, and it does not influence the disease progression or treatment. Thus, bias arising due to confounding and reverse causality is avoided in causal inferences. Several MR analyses have explored the causal relationship between TL and the risk of cancer, suggesting that longer TL is associated with an increased risk of glioma, lung adenocarcinoma, and melanoma.^33^, ^34^, ^35^ The association strength is significantly different in different cancer types (OR, 1.31–5.27).^33^ However, TL is not always associated with cancer risk. For example, testicular germ cell tumor, lung squamous cell carcinoma, and head and neck cancer are not associated with TL.^36^, ^37^

When we performed MR analysis using the above‐mentioned five methods, each of which offers unique merits and performed sensitivity analyses to ensure the robustness of the results, no linear causal relationship was observed between the TL and HCC risk. Previous observational studies showed an association between TL and HCC risk in the Asian population. A case–control study (240 HCC cases, 240 healthy controls) reported that, when compared to the healthy controls, individuals with a relatively long TL had a significantly increased risk of HBV‐related HCC after adjusting for factors such as age and sex (adjusted OR, 7.28; 95% CI, 4.46–11.88).^11^ A similar conclusion (OR: 3.22, 95% CI: 2.01–5.17) was obtained in another case–control study (152 patients with HCC, 184 healthy controls).^12^ However, contrary to these reports, the TL of HCC cases in the Europeans were significantly shorter than that in controls (40 cases, 64 controls, *p* = 0.0006).^38^ Based on a more rigorous study design, our conclusions differed from those of the past studies. This observation can be attributed to the inherent limitations of the methodology followed for an observational study, where, a causal relationship could not be demonstrated. Furthermore, a small sample size used in these observational studies may not be sufficient to reach a convincing conclusion.

In addition, an MR analysis conducted on TL and HBV‐related HCC risk (1538 HBV‐positive cases and 1465 HBV‐positive controls) in the Asian population reported individual‐level data for analysis along with a U‐shaped relationship.^14^ However, this finding does not contradict our findings. First, there may be multiple etiologies of HCC in addition to HBV in the data we used. It has been reported that HBV may induce cell senescence because chronic HBV infection is related to the arrest of G1 cell cycle of hepatocytes and the acceleration of hepatocyte senescence.^39^ According the assumptions of MR, HBV may be a confounder. Other causes of HCC, including alcohol and HCV, have not been reported in relation to TL. Hence, the causality of TL on HCC of unclear cause may be different from that of HBV‐related HCC. Second, the above report reported a U‐shaped relationship, which is a non‐linear causal relationship between TL and HCC, which is consistent with our results of no linear causality essentially. Furthermore, the previously published MR analyses are based on the Asian population and are inadequate for use in extrapolating the conclusions to the remaining global population.

Our study offers several advantages. The nine SNPs in Asians and 98 SNPs in Europeans stringently selected were all independent and rigidly associated with TL, which avoids the effect of linkage disequilibrium on the causal estimate. Considering that both MR analyses were based on either all Asians or all Europeans, bias arising due to population stratification was minimized. Therefore, the SNPs selected in this study and the corresponding research results are significant and reliable. Similar conclusions obtained across two different ethnic groups despite their environmental differences increase the generalization of our conclusions at the global level.

Although the methodology of MR analysis is valuable in causal inference, it has only a few limitations. The MR analysis assumed a linear relationship between the two elements being studied in the causal relationship, even when the possibilities of other nonlinear relationships such as J or U‐shape existed, thereby weakening the power of causal inference. Owing to the limitation of public data, we could not explore the non‐linear relationships of TL and HCC. Furthermore, subgroup analyses based on gender, age, and comorbidities were limited and caution was exercised when extending the conclusions to the individual level because summary‐level statistics were obtained.

In conclusion, our two‐sample MR analysis revealed no significant linear causal relationship between human TL and the risk of HCC in the Asian and European populations.

## AUTHOR CONTRIBUTIONS

**Chenglei Yang:** Conceptualization (equal); investigation (equal); methodology (equal); project administration (equal); supervision (equal); validation (equal); writing – review and editing (equal). **Xi Wu:** Data curation (equal); formal analysis (equal); investigation (equal); methodology (equal); resources (equal); software (equal); visualization (equal); writing – original draft (equal); writing – review and editing (equal). **Siyu Chen:** Data curation (equal); investigation (equal); methodology (equal); visualization (equal). **Bangde Xiang:** Funding acquisition (equal); project administration (equal); supervision (equal); validation (equal).

## FUNDING INFORMATION

This work was supported by the High‐level Innovation Team and Outstanding Scholar Program in Guangxi Colleges and Universities, Innovation Project of Guangxi Graduate Education (No. YCBZ2022089); College Students' Innovation and Entrepreneurship Training Program (S202210598055); and Guangxi Key Research and Development Program (AA18221001, AB18050020).

## CONFLICT OF INTEREST STATEMENT

The authors declare no conflicts of interest.

## STATEMENT OF ETHICAL APPROVAL

The data used in our study were obtained from published studies or public databases, and no original data were sourced from patients. Therefore, no ethics committee approval was required.

## Supporting information


Figure S1.
Figure S2.

Table S1.

## Data Availability

Statistics supporting the findings of this study are all available from PubMed (https://pubmed.ncbi.nlm.nih.gov/) the IEU GWAS public database (https://gwas.mrcieu.ac.uk/datasets/).
