# Supplementary material for: Association between telomere length and hepatocellular carcinoma risk: A Mendelian randomization study
Source: Cancer Med. 2023 Mar 7;12(8):9937–44. doi: 10.1002/cam4.5702 (PMC10166926; doi:10.1002/cam4.5702)
Supplement: Supplementary file 1 — Figure S1. Figure S2. [file CAM4-12-9937-s001.zip › CAM4_5702_Legends to Supplementary Figures.docx]

**Supplementary Figure 1.** The leave-one-out analysis of causal estimates of TL on HCC risk in Asians. Black lines represent the pooled effect and 95% CI of the remaining SNPs after a specific SNP was removed, and the red lines represent the pooled effect and 95% CI of all SNPs. Exposure, telomere length; outcome, hepatocellular carcinoma. MR, Mendelian randomization; TL, telomere length; HCC, hepatocellular carcinoma; CI, confidence interval; SNP, single nucleotide polymorphism.

**Supplementary Figure 2.** The leave-one-out analysis of the causal estimates of TL on HCC risk in the Europeans. The black lines represent the pooled effect and 95% CI of the remaining SNPs after a specific SNP was removed, and the red lines represent the pooled effect and 95% CI of all SNPs. Exposure, telomere length; outcome, hepatocellular carcinoma. MR, Mendelian randomization; TL, telomere length; HCC, hepatocellular carcinoma; CI, confidence interval; SNP, single nucleotide polymorphism.
