# Supplementary figures and images for: Association between telomere length and hepatocellular carcinoma risk: A Mendelian randomization study
Source: Cancer Med. 2023 Mar 7;12(8):9937–44. doi: 10.1002/cam4.5702 (PMC10166926; doi:10.1002/cam4.5702)

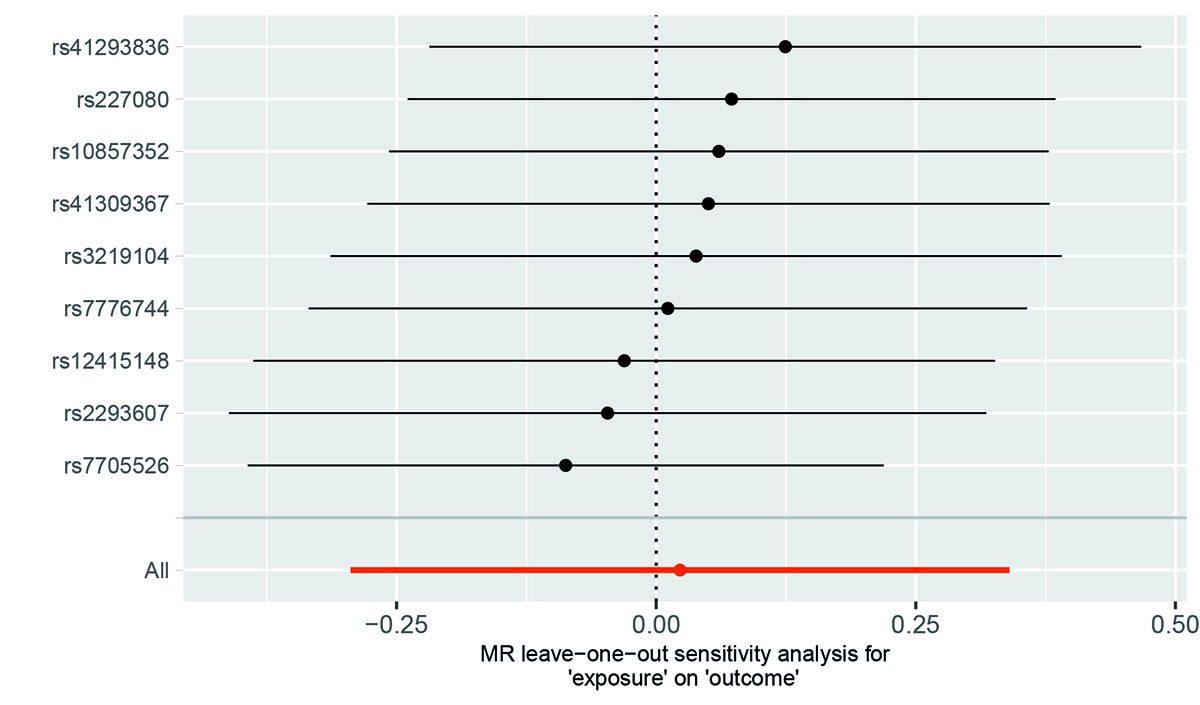

Supplement: Supplementary file 1 — Figure S1. Figure S2. [file CAM4-12-9937-s001.zip › cam45702-sup-0001-FigureS1.jpg]

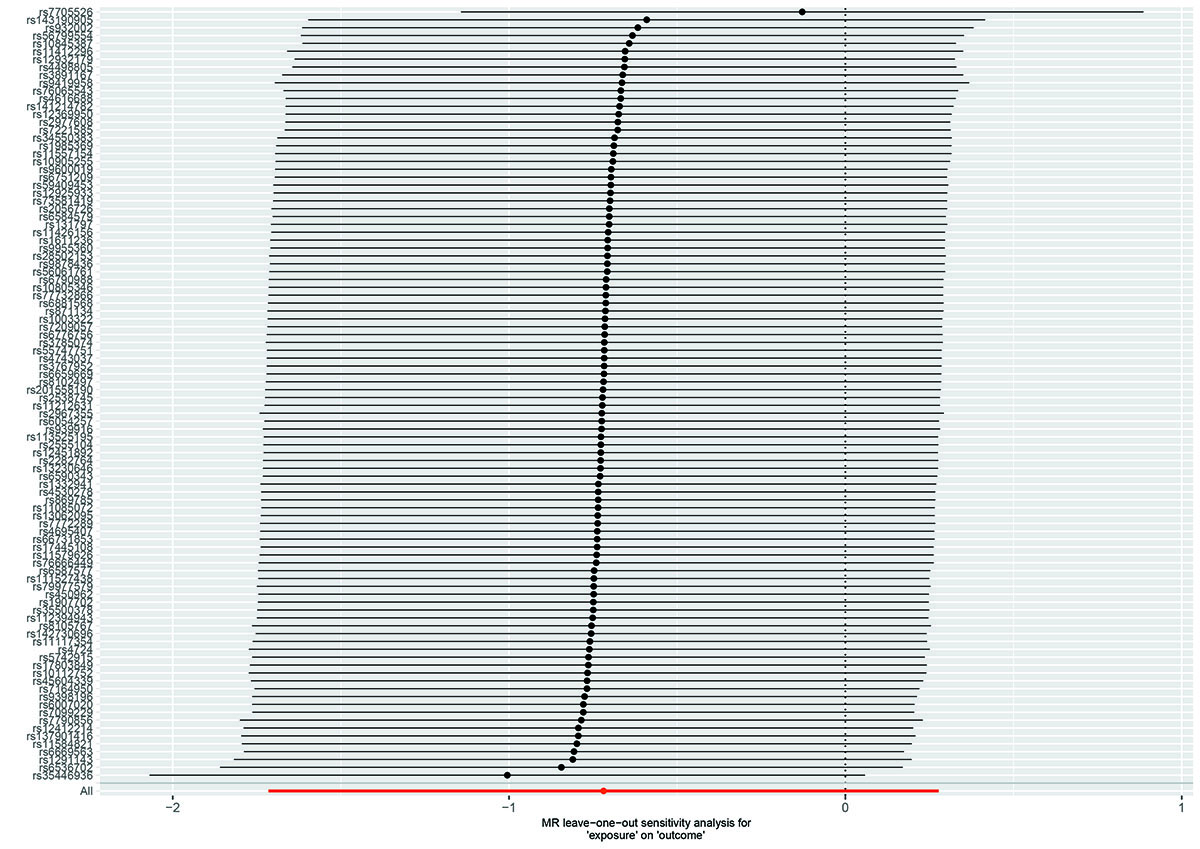

Supplement: Supplementary file 1 — Figure S1. Figure S2. [file CAM4-12-9937-s001.zip › cam45702-sup-0002-FigureS2.jpg]
