# Supplementary material for: Association between telomere length and hepatocellular carcinoma risk: A Mendelian randomization study
Source: Cancer Med. 2023 Mar 7;12(8):9937–44. doi: 10.1002/cam4.5702 (PMC10166926; doi:10.1002/cam4.5702)
Supplement: Supplementary file 2 — Table S1. [file CAM4-12-9937-s002.docx]

| **Supplementary Table 1. Characteristics of genetic variants associated with telomere length** | | | | | | | | | | | |  |
| --- | --- | --- | --- | --- | --- | --- | --- | --- | --- | --- | --- | --- |
| Population | SNP | POS | EA | OA | SNP-TL | | | | SNP-HCC | | |  |
|  |  |  |  |  | beta | SE | P-value | F | beta | SE | P-value |  |
| Asian | rs3219104 | chr1:226562621 | A | C | -7.40E-02 | 9.00E-03 | 2.23E-16 | 67.60 | 1.09E-02 | 3.37E-02 | 7.46E-01 |  |
| Asian | rs2293607 | chr3:169482335 | T | C | -1.20E-01 | 9.00E-03 | 7.57E-39 | 177.76 | -3.42E-02 | 3.44E-02 | 3.20E-01 |  |
| Asian | rs10857352 | chr4:164101482 | A | G | -6.40E-02 | 1.10E-02 | 4.85E-09 | 33.85 | 5.54E-02 | 4.18E-02 | 1.85E-01 |  |
| Asian | rs7705526 | chr5:1285974 | C | A | -1.18E-01 | 9.00E-03 | 2.61E-38 | 171.89 | -7.28E-02 | 3.92E-02 | 6.32E-02 |  |
| Asian | rs7776744 | chr7:124599749 | A | G | -5.80E-02 | 9.00E-03 | 2.51E-10 | 41.53 | -1.42E-02 | 3.40E-02 | 6.77E-01 |  |
| Asian | rs227080 | chr11:108247888 | A | G | -6.00E-02 | 9.00E-03 | 1.87E-10 | 44.44 | 4.95E-02 | 3.35E-02 | 1.40E-01 |  |
| Asian | rs12415148 | chr10:105680586 | T | C | -2.04E-01 | 2.00E-02 | 2.78E-25 | 104.03 | -6.12E-02 | 6.65E-02 | 3.58E-01 |  |
| Asian | rs41293836 | chr14:24721327 | C | T | -2.33E-01 | 1.70E-02 | 2.47E-42 | 187.83 | 8.58E-02 | 6.75E-02 | 2.03E-01 |  |
| Asian | rs41309367 | chr20:62309554 | C | T | -5.80E-02 | 1.00E-02 | 1.16E-08 | 33.64 | 4.13E-02 | 4.02E-02 | 3.04E-01 |  |
| European | rs3767952 | chr1:41231032 | A | G | 1.34E-02 | 2.39E-03 | 1.80E-08 | 31.70 | -1.86E-02 | 1.31E-01 | 8.90E-01 |  |
| European | rs4498805 | chr1:110910397 | T | G | 1.51E-02 | 2.00E-03 | 5.70E-14 | 56.49 | -2.15E-01 | 1.09E-01 | 5.00E-02 |  |
| European | rs932002 | chr1:226577306 | T | C | -4.02E-02 | 2.80E-03 | 7.30E-47 | 206.67 | 2.67E-01 | 1.52E-01 | 7.80E-02 |  |
| European | rs66731853 | chr1:20916238 | A | G | -1.78E-02 | 2.15E-03 | 1.50E-16 | 68.11 | -4.74E-02 | 1.17E-01 | 6.80E-01 |  |
| European | rs11584821 | chr1:114419489 | T | C | -3.07E-02 | 2.64E-03 | 3.00E-31 | 135.19 | -1.95E-01 | 1.43E-01 | 1.70E-01 |  |
| European | rs6659669 | chr1:185315067 | T | C | -1.17E-02 | 2.05E-03 | 1.10E-08 | 32.57 | 1.33E-02 | 1.11E-01 | 9.10E-01 |  |
| European | rs2977608 | chr1:768253 | C | A | 1.29E-02 | 2.34E-03 | 3.00E-08 | 30.69 | -2.33E-01 | 1.28E-01 | 6.90E-02 |  |
| European | rs6669563 | chr1:32279629 | A | G | 1.82E-02 | 2.02E-03 | 2.10E-19 | 81.11 | 2.29E-01 | 1.10E-01 | 3.70E-02 |  |
| European | rs11579626 | chr1:146741960 | C | A | 2.65E-02 | 3.58E-03 | 1.30E-13 | 54.92 | 1.05E-01 | 1.96E-01 | 5.90E-01 |  |
| European | rs6587577 | chr1:151402045 | G | A | -1.82E-02 | 2.64E-03 | 4.80E-12 | 47.75 | -1.21E-01 | 1.45E-01 | 4.00E-01 |  |
| European | rs6751209 | chr2:43588302 | C | T | -1.40E-02 | 2.48E-03 | 1.60E-08 | 31.96 | 1.30E-01 | 1.36E-01 | 3.40E-01 |  |
| European | rs17803849 | chr2:210673445 | T | C | 2.73E-02 | 2.03E-03 | 4.20E-41 | 180.27 | 6.49E-02 | 1.11E-01 | 5.60E-01 |  |
| European | rs2555104 | chr2:17841243 | C | A | -1.40E-02 | 2.03E-03 | 6.60E-12 | 47.14 | -1.93E-02 | 1.10E-01 | 8.60E-01 |  |
| European | rs77732866 | chr2:58979879 | A | G | 1.78E-02 | 2.91E-03 | 9.20E-10 | 37.50 | -5.16E-02 | 1.58E-01 | 7.40E-01 |  |
| European | rs9878436 | chr3:138244400 | T | C | -1.43E-02 | 2.02E-03 | 1.20E-12 | 50.49 | 4.96E-02 | 1.10E-01 | 6.50E-01 |  |
| European | rs35446936 | chr3:169486508 | A | G | -9.40E-02 | 2.33E-03 | 1.00E-200 | 1628.81 | -1.09E-01 | 1.27E-01 | 3.90E-01 |  |
| European | rs112394943 | chr3:197842892 | C | T | -1.99E-02 | 2.82E-03 | 1.60E-12 | 49.90 | -1.49E-01 | 1.55E-01 | 3.40E-01 |  |
| European | rs13062095 | chr3:101267385 | C | T | 1.39E-02 | 2.14E-03 | 9.70E-11 | 41.87 | 6.03E-02 | 1.16E-01 | 6.00E-01 |  |
| European | rs4616688 | chr3:160042459 | T | G | -1.73E-02 | 2.00E-03 | 4.50E-18 | 75.09 | 1.58E-01 | 1.09E-01 | 1.50E-01 |  |
| European | rs6790988 | chr3:170263320 | G | A | 1.46E-02 | 2.28E-03 | 1.80E-10 | 40.70 | -4.39E-02 | 1.24E-01 | 7.20E-01 |  |
| European | rs869785 | chr3:24347800 | C | T | -1.47E-02 | 2.13E-03 | 4.40E-12 | 47.92 | -5.03E-02 | 1.16E-01 | 6.60E-01 |  |
| European | rs6776756 | chr3:128215821 | A | G | -1.74E-02 | 2.04E-03 | 1.10E-17 | 73.30 | 2.18E-02 | 1.11E-01 | 8.40E-01 |  |
| European | rs11426156 | chr3:128318179 | T | TA | -1.16E-02 | 2.07E-03 | 2.20E-08 | 31.31 | 7.11E-02 | 1.13E-01 | 5.30E-01 |  |
| European | rs35500378 | chr4:122729413 | CACTT | C | 1.45E-02 | 2.06E-03 | 2.00E-12 | 49.51 | 9.93E-02 | 1.12E-01 | 3.70E-01 |  |
| European | rs10805346 | chr4:9920347 | C | T | 1.17E-02 | 2.02E-03 | 7.00E-09 | 33.54 | -3.90E-02 | 1.10E-01 | 7.20E-01 |  |
| European | rs6536702 | chr4:164028105 | A | G | 5.34E-02 | 2.39E-03 | 9.40E-111 | 500.01 | 1.21E-01 | 1.30E-01 | 3.50E-01 |  |
| European | rs871134 | chr4:7044380 | T | C | -1.83E-02 | 2.03E-03 | 1.70E-19 | 81.55 | 2.84E-02 | 1.11E-01 | 8.00E-01 |  |
| European | rs4695407 | chr4:48843372 | G | A | 1.42E-02 | 2.00E-03 | 1.50E-12 | 50.10 | 5.54E-02 | 1.09E-01 | 6.10E-01 |  |
| European | rs2282764 | chr4:2255063 | G | A | -2.24E-02 | 2.89E-03 | 9.30E-15 | 60.04 | -2.55E-02 | 1.60E-01 | 8.70E-01 |  |
| European | rs7705526 | chr5:1285974 | A | C | 7.76E-02 | 2.16E-03 | 1.00E-200 | 1289.26 | -4.52E-01 | 1.18E-01 | 1.30E-04 |  |
| European | rs6881568 | chr5:1670265 | A | C | 1.69E-02 | 2.08E-03 | 3.70E-16 | 66.38 | -3.26E-02 | 1.13E-01 | 7.70E-01 |  |
| European | rs55747751 | chr5:132397351 | A | G | -2.12E-02 | 3.75E-03 | 1.70E-08 | 31.82 | 3.15E-02 | 2.01E-01 | 8.80E-01 |  |
| European | rs141214782 | chr5:78954683 | TTATC | T | -2.47E-02 | 3.36E-03 | 2.00E-13 | 53.99 | 2.99E-01 | 1.88E-01 | 1.10E-01 |  |
| European | rs1611236 | chr6:29748690 | A | G | -1.60E-02 | 2.13E-03 | 6.10E-14 | 56.33 | 5.43E-02 | 1.17E-01 | 6.40E-01 |  |
| European | rs142730696 | chr6:26360448 | TTTTTC | T | 2.17E-02 | 3.02E-03 | 6.30E-13 | 51.74 | 1.73E-01 | 1.63E-01 | 2.90E-01 |  |
| European | rs7772289 | chr6:28674322 | T | G | 1.75E-02 | 2.00E-03 | 1.70E-18 | 76.99 | 3.68E-02 | 1.09E-01 | 7.40E-01 |  |
| European | rs201558190 | chr6:29877484 | C | T | -1.82E-02 | 2.17E-03 | 6.40E-17 | 69.84 | 7.47E-03 | 1.19E-01 | 9.50E-01 |  |
| European | rs9398196 | chr6:109601554 | G | A | -1.44E-02 | 2.01E-03 | 9.50E-13 | 50.94 | -1.85E-01 | 1.09E-01 | 9.10E-02 |  |
| European | rs2538745 | chr7:76310784 | C | T | -1.29E-02 | 2.06E-03 | 3.10E-10 | 39.62 | -3.72E-03 | 1.11E-01 | 9.70E-01 |  |
| European | rs1985369 | chr7:159119220 | G | A | -3.12E-02 | 3.01E-03 | 3.60E-25 | 107.40 | 1.32E-01 | 1.65E-01 | 4.20E-01 |  |
| European | rs13230646 | chr7:23930316 | C | T | -1.73E-02 | 2.32E-03 | 8.90E-14 | 55.60 | -2.26E-02 | 1.26E-01 | 8.60E-01 |  |
| European | rs2056726 | chr7:99780283 | A | G | -2.28E-02 | 2.44E-03 | 7.90E-21 | 87.63 | 7.00E-02 | 1.32E-01 | 5.90E-01 |  |
| European | rs7790856 | chr7:124459852 | T | C | -4.37E-02 | 2.21E-03 | 1.80E-87 | 393.04 | -5.70E-02 | 1.20E-01 | 6.40E-01 |  |
| European | rs10112752 | chr8:73958718 | A | G | -2.88E-02 | 2.03E-03 | 9.50E-46 | 201.56 | -6.21E-02 | 1.10E-01 | 5.70E-01 |  |
| European | rs11557154 | chr9:34107505 | T | C | -3.44E-02 | 2.99E-03 | 1.10E-30 | 132.56 | 1.18E-01 | 1.64E-01 | 4.70E-01 |  |
| European | rs4743037 | chr9:109639970 | T | C | 1.48E-02 | 2.38E-03 | 5.10E-10 | 38.62 | -1.95E-02 | 1.31E-01 | 8.80E-01 |  |
| European | rs7099229 | chr10:96134685 | A | G | -1.53E-02 | 2.24E-03 | 8.40E-12 | 46.66 | -2.38E-01 | 1.23E-01 | 5.40E-02 |  |
| European | rs12412214 | chr10:101276256 | A | G | -2.45E-02 | 2.23E-03 | 3.40E-28 | 121.22 | -1.67E-01 | 1.21E-01 | 1.70E-01 |  |
| European | rs6584579 | chr10:105645725 | G | A | 1.15E-02 | 2.05E-03 | 2.00E-08 | 31.53 | -8.47E-02 | 1.12E-01 | 4.50E-01 |  |
| European | rs9419958 | chr10:105675946 | C | T | -8.10E-02 | 2.94E-03 | 2.60E-167 | 760.03 | 1.28E-01 | 1.62E-01 | 4.30E-01 |  |
| European | rs10905255 | chr10:5870267 | T | G | -1.82E-02 | 2.03E-03 | 2.60E-19 | 80.74 | 9.02E-02 | 1.11E-01 | 4.20E-01 |  |
| European | rs939916 | chr11:202253 | A | G | 2.42E-02 | 2.17E-03 | 6.60E-29 | 124.47 | -2.77E-03 | 1.18E-01 | 9.80E-01 |  |
| European | rs11212631 | chr11:108304509 | C | T | -1.93E-02 | 2.57E-03 | 4.70E-14 | 56.86 | -1.99E-03 | 1.39E-01 | 9.90E-01 |  |
| European | rs6590343 | chr11:128500215 | G | A | 1.22E-02 | 2.01E-03 | 1.50E-09 | 36.52 | 3.32E-02 | 1.09E-01 | 7.60E-01 |  |
| European | rs12369950 | chr12:24762109 | C | T | -1.78E-02 | 2.90E-03 | 8.00E-10 | 37.75 | 2.75E-01 | 1.58E-01 | 8.30E-02 |  |
| European | rs79977579 | chr12:54694560 | A | C | 2.82E-02 | 3.43E-03 | 2.30E-16 | 67.29 | 1.27E-01 | 1.84E-01 | 4.90E-01 |  |
| European | rs10845387 | chr12:11757743 | A | G | -1.41E-02 | 2.09E-03 | 1.50E-11 | 45.48 | 3.04E-01 | 1.15E-01 | 8.00E-03 |  |
| European | rs17445108 | chr12:57082058 | A | G | -1.69E-02 | 3.01E-03 | 2.00E-08 | 31.50 | -1.10E-01 | 1.61E-01 | 5.00E-01 |  |
| European | rs1907702 | chr12:88955469 | A | G | 1.50E-02 | 2.43E-03 | 5.90E-10 | 38.34 | 1.36E-01 | 1.32E-01 | 3.00E-01 |  |
| European | rs76666449 | chr12:120904895 | C | T | 2.95E-02 | 3.33E-03 | 8.20E-19 | 78.46 | 7.82E-02 | 1.80E-01 | 6.60E-01 |  |
| European | rs1332941 | chr13:41695100 | G | A | 2.57E-02 | 2.73E-03 | 5.90E-21 | 88.21 | 3.77E-02 | 1.50E-01 | 8.00E-01 |  |
| European | rs9600019 | chr13:73317585 | T | C | 1.27E-02 | 2.13E-03 | 2.40E-09 | 35.59 | -1.09E-01 | 1.16E-01 | 3.50E-01 |  |
| European | rs73581419 | chr14:21941148 | T | C | 2.30E-02 | 3.24E-03 | 1.30E-12 | 50.27 | -1.23E-01 | 1.75E-01 | 4.80E-01 |  |
| European | rs113525195 | chr14:23499321 | A | C | -1.24E-02 | 2.24E-03 | 3.10E-08 | 30.64 | -3.01E-02 | 1.22E-01 | 8.10E-01 |  |
| European | rs45604339 | chr14:65543102 | T | C | -2.04E-02 | 2.11E-03 | 4.30E-22 | 93.39 | -1.16E-01 | 1.15E-01 | 3.10E-01 |  |
| European | rs137901416 | chr14:73418095 | A | G | 4.57E-02 | 3.32E-03 | 4.70E-43 | 189.24 | 1.86E-01 | 1.80E-01 | 3.00E-01 |  |
| European | rs34550383 | chr14:91971787 | C | CT | -1.92E-02 | 2.00E-03 | 8.60E-22 | 92.01 | 9.80E-02 | 1.10E-01 | 3.70E-01 |  |
| European | rs5742915 | chr15:74336633 | C | T | 1.93E-02 | 2.03E-03 | 1.60E-21 | 90.85 | 1.01E-01 | 1.09E-01 | 3.60E-01 |  |
| European | rs7164950 | chr15:56775385 | G | A | 1.29E-02 | 2.04E-03 | 2.30E-10 | 40.21 | 1.86E-01 | 1.11E-01 | 9.40E-02 |  |
| European | rs11412296 | chr15:50366116 | T | TA | 3.32E-02 | 2.34E-03 | 1.40E-45 | 200.79 | -1.52E-01 | 1.27E-01 | 2.30E-01 |  |
| European | rs2967355 | chr16:82200103 | C | A | -4.62E-02 | 2.39E-03 | 4.00E-83 | 373.10 | 2.53E-02 | 1.30E-01 | 8.40E-01 |  |
| European | rs12925933 | chr16:90141355 | C | A | -1.47E-02 | 2.14E-03 | 7.00E-12 | 47.03 | 8.93E-02 | 1.17E-01 | 4.40E-01 |  |
| European | rs450962 | chr16:28413517 | G | A | 1.43E-02 | 2.45E-03 | 5.90E-09 | 33.87 | 1.40E-01 | 1.32E-01 | 2.90E-01 |  |
| European | rs3785074 | chr16:69406986 | G | A | 2.39E-02 | 2.20E-03 | 2.60E-27 | 117.17 | -2.26E-02 | 1.21E-01 | 8.50E-01 |  |
| European | rs76065543 | chr16:74678063 | T | C | 3.43E-02 | 2.91E-03 | 4.20E-32 | 139.08 | -1.78E-01 | 1.58E-01 | 2.60E-01 |  |
| European | rs12932179 | chr16:9072085 | G | A | -1.36E-02 | 2.03E-03 | 1.80E-11 | 45.16 | 2.45E-01 | 1.11E-01 | 2.70E-02 |  |
| European | rs11117354 | chr16:88092092 | C | T | 2.33E-02 | 2.20E-03 | 3.40E-26 | 112.10 | 8.80E-02 | 1.20E-01 | 4.60E-01 |  |
| European | rs56061761 | chr16:70187811 | A | G | -2.04E-02 | 2.23E-03 | 6.90E-20 | 83.35 | 4.74E-02 | 1.21E-01 | 7.00E-01 |  |
| European | rs4724 | chr17:7760397 | A | G | -5.47E-02 | 3.12E-03 | 9.80E-69 | 307.00 | -5.63E-02 | 1.74E-01 | 7.50E-01 |  |
| European | rs12451892 | chr17:2247982 | C | T | -1.16E-02 | 2.08E-03 | 2.20E-08 | 31.31 | -2.99E-02 | 1.13E-01 | 7.90E-01 |  |
| European | rs7209057 | chr17:65705530 | A | G | 1.18E-02 | 2.03E-03 | 5.70E-09 | 33.94 | -2.35E-02 | 1.11E-01 | 8.30E-01 |  |
| European | rs59409453 | chr17:1666218 | G | A | 2.02E-02 | 2.30E-03 | 1.60E-18 | 77.12 | -8.38E-02 | 1.25E-01 | 5.00E-01 |  |
| European | rs111527438 | chr17:29252703 | C | T | 1.25E-02 | 2.11E-03 | 3.10E-09 | 35.09 | 1.15E-01 | 1.14E-01 | 3.10E-01 |  |
| European | rs7221585 | chr17:76195153 | T | C | 1.43E-02 | 2.47E-03 | 6.70E-09 | 33.63 | -2.35E-01 | 1.36E-01 | 8.40E-02 |  |
| European | rs56799554 | chr17:41456413 | G | A | -2.60E-02 | 2.68E-03 | 3.00E-22 | 94.07 | 3.13E-01 | 1.47E-01 | 3.30E-02 |  |
| European | rs9955360 | chr18:78008334 | A | C | -1.90E-02 | 3.00E-03 | 2.20E-10 | 40.30 | 8.58E-02 | 1.66E-01 | 6.00E-01 |  |
| European | rs3891167 | chr18:658423 | G | A | -4.26E-02 | 2.40E-03 | 1.20E-70 | 315.78 | 1.22E-01 | 1.30E-01 | 3.50E-01 |  |
| European | rs11085072 | chr19:4368142 | T | C | -1.32E-02 | 2.37E-03 | 2.60E-08 | 31.00 | -7.45E-02 | 1.28E-01 | 5.60E-01 |  |
| European | rs4530278 | chr19:33752994 | T | G | 1.39E-02 | 2.06E-03 | 1.50E-11 | 45.54 | 5.03E-02 | 1.12E-01 | 6.50E-01 |  |
| European | rs8105767 | chr19:22215441 | G | A | 3.28E-02 | 2.20E-03 | 2.50E-50 | 222.56 | 4.17E-02 | 1.21E-01 | 7.30E-01 |  |
| European | rs8102497 | chr19:57370055 | A | G | -1.50E-02 | 2.02E-03 | 1.40E-13 | 54.71 | 1.02E-02 | 1.10E-01 | 9.30E-01 |  |
| European | rs1291143 | chr20:35525640 | C | A | 4.93E-02 | 2.80E-03 | 1.80E-69 | 310.39 | 1.45E-01 | 1.54E-01 | 3.50E-01 |  |
| European | rs6054257 | chr20:66370 | A | G | -1.42E-02 | 2.48E-03 | 1.10E-08 | 32.71 | -1.96E-02 | 1.35E-01 | 8.80E-01 |  |
| European | rs143190905 | chr20:62291767 | T | G | -7.24E-02 | 3.69E-03 | 1.60E-85 | 384.08 | 3.54E-01 | 2.05E-01 | 8.50E-02 |  |
| European | rs28502153 | chr22:17469049 | A | C | -2.16E-02 | 2.06E-03 | 1.20E-25 | 109.64 | 4.34E-02 | 1.13E-01 | 7.00E-01 |  |
| European | rs6007020 | chr22:45790132 | C | T | 1.45E-02 | 2.10E-03 | 4.80E-12 | 47.78 | 2.14E-01 | 1.14E-01 | 6.10E-02 |  |
| European | rs131797 | chr22:50971631 | T | TAAAAA | 2.44E-02 | 2.37E-03 | 6.80E-25 | 106.17 | -6.36E-02 | 1.29E-01 | 6.20E-01 |  |
| European | rs1003322 | chr22:51072289 | A | C | 1.42E-02 | 2.48E-03 | 1.00E-08 | 32.78 | -3.26E-02 | 1.35E-01 | 8.10E-01 |  |
| SNP, single nucleotide polymorphisms; POS, position of SNP, including chromosome number and base pair information; EA, effect allele; OA, other allele; SE, standard error; F, F-statistics; TL, telomere length; HCC, hepatocellular carcinoma. | | | | | | | | | | | |  |
|  |  |  |  |  |  |  |  |  |  |  |  |  |
